# Editorial: Climate change anxiety

**DOI:** 10.3389/fpsyg.2026.1812351

**Published:** 2026-03-13

**Authors:** Matteo Innocenti, Alessandro von Gal, Laura Piccardi, Joshua M. Carlson, Francisco Sampaio, John Jamir Benzon Aruta

**Affiliations:** 1 Department of Life Sciences and Public Health, Università Cattolica del Sacro Cuore, Rome, Italy; 2 Department of Psychology, Faculty of Medicine and Psychology, Sapienza University of Rome, Rome, Italy; 3 IRCCS San Raffaele Cassino, Cassino, Italy; 4 Department of Psychological Science, Northern Michigan University, Marquette, MI, United States; 5 RISE-Health, Nursing School, University of Porto, Porto, Portugal; 6 De La Salle University, Manila, Philippines

**Keywords:** adolescents, climate change anxiety, climate emotions, climate worry, eco-anxiety, mental health, pro-environmental behavior, psychometrics

## 1 Introduction

Over the past decade, climate change anxiety has moved from a mainly conceptual and interdisciplinary concern to a rapidly expanding empirical field within environmental psychology, mental health, and behavioral science. Early scholarship helped establish that climate-related anxiety can be an intelligible and often non-clinical response to real ecological threats, while also recognizing that, in some cases, distress may become persistent, overwhelming, and functionally impairing. As the field developed, psychometric instruments and cross-cultural validation studies accelerated research output, but also made visible ongoing heterogeneity in definitions and operationalizations, including the boundaries between climate anxiety, climate worry, and broader climate emotions. More recently, large-scale population studies and quantitative syntheses have reinforced the public health relevance of this topic and highlighted the importance of conceptually precise, culturally sensitive, and justice-attentive approaches to assessment and interpretation.

Climate change anxiety has gained prominence as a construct that captures distress responses to perceived and anticipated climate threats. The term is used heterogeneously in the literature, sometimes referring to worry and heightened concern, and other times referring to more impairing forms of anxiety that affect sleep, concentration, decision-making, and everyday functioning. The present Research Topic was assembled to advance conceptual clarity and empirical precision, while explicitly avoiding the pathologization of proportionate distress in the face of real-world risks.

**Figure 1:**
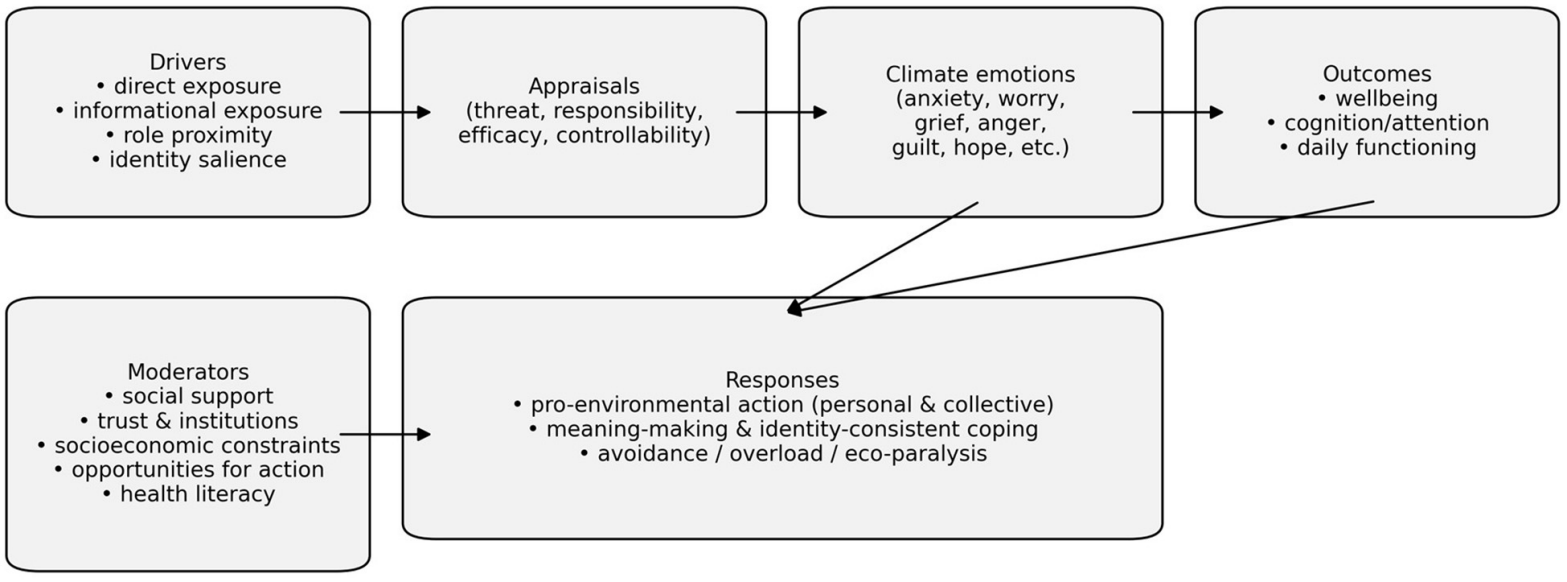
Conceptual synthesis of the pathways linking climate-related drivers and appraisals to climate emotions (including climate change anxiety), functional outcomes, and behavioral responses. The model emphasizes moderators such as social support, trust, socioeconomic constraints, opportunities for action, and health literacy. This figure is intended as an integrative framework based on themes across the contributions in this Research Topic (it contains no original data).

This Research Topic includes 17 articles published across Frontiers journals (including Frontiers in Psychology, Frontiers in Climate, and Frontiers in Sustainable Cities), reflecting the interdisciplinary nature of climate-related distress research. Contributions span psychometric evaluation and validation, meta-analytic synthesis, bibliometric mapping, cross-sectional population studies, and qualitative work on lived experience and coping. Together, they offer a snapshot of a field that is expanding rapidly while still consolidating shared conceptual and methodological standards. An integrative conceptual synthesis of the pathways linking climate-related drivers, emotions, and behavioral responses is presented in Figure 1.

## 2 Key themes across the Research Topic

### 2.1 Conceptualization and measurement: clarifying what is being assessed

A foundational step in cumulative science is understanding how the literature is developing and where major emphases and gaps lie. *Emotional climate: a bibliometric analysis of the psychological consequences of climate change* provides a structured map of research output on the psychological consequences of climate change, helping to situate climate change anxiety within a broader and fast-growing field (Momenpour and Choobchian).

Several contributions focus on strengthening measurement and construct boundaries. *Eco-emotions: validation of the multi-dimensional inventory of climate emotions in an Australian sample* validates a multi-dimensional measure designed to capture a range of climate-related emotions concurrently, supporting work that moves beyond single-emotion approaches (Rice et al.).

Two adolescent-focused studies extend the psychometric evidence base for measuring climate anxiety in youth: *Exploring eco-anxiety in Italian adolescents: psychometric evaluation of the Climate Change Anxiety Scale and theoretical insights into the association with pro-environmental attitudes* evaluates the Climate Change Anxiety Scale in Italian adolescents and discusses links with pro-environmental attitudes (Innocenti et al.), while *Psychometric properties of the Spanish version of the climate anxiety scale in Spanish-speaking adolescents* examines the psychometric performance of a Spanish version in a large adolescent sample and includes evidence relevant to comparability across groups (Jimenez-Vazquez et al.).

At the level of climate-related worry, *Reliability generalization meta-analysis of the Climate Change Worry Scale* synthesizes reliability evidence across studies, highlighting the importance of reporting and evaluating measurement properties in each sample and context (Gezer et al.).

Finally, construct refinement is addressed directly in *Eco-anxiety or simply eco-worry? Incremental validity study in a representative Spanish sample* (Vecina et al.), which interrogates whether “eco-anxiety” provides explanatory value beyond “eco-worry,” a question that is central to both theory building and careful interpretation in applied contexts.

### 2.2 From climate-related distress to functioning and mental health outcomes

A distinctive contribution of the Research Topic is the move beyond self-reported distress toward functional and cognitive correlates. *Climate anxiety impairs sustained attention: objective evidence of a cognitive cost* provides objective evidence that higher climate anxiety is associated with poorer sustained attention performance, even when controlling for broader symptoms such as general anxiety and depression (Denkova et al.).

Population studies in adolescents and young adults further examine distress and impairment in relation to contextual and individual factors. *Climate change distress and impairment among adolescents in Germany* investigates climate change distress and impairment among adolescents in Germany and explores associations with a range of demographic and literacy-related factors (König et al.). In a university context, *The impact of climate change experience on Palestinian university students' mental health: a cross-sectional study* links reported climate change experiences with mental health indicators and academically relevant impacts, underscoring that climate-related stressors may be intertwined with educational functioning (Ahmead et al.).

The Research Topic also broadens the discussion to specific settings and vulnerabilities. *Impact factors of Arctic research stations on the mental health of team members* examines how station environment and design features may relate to team members' mental health, highlighting the relevance of place- and occupation-based contexts (Li and Zou). A gender- and exposure-attuned lens is offered by *Exploring eco-anxiety among women amid climate-induced heat: a comprehensive review* (Gayathiri et al.), which synthesizes evidence and gaps regarding eco-anxiety among women in the context of climate-induced heat and calls for more globally inclusive and longitudinal research.

### 2.3 Identity, roles, and meaning-making in climate anxiety

Several studies foreground identity and role proximity as key lenses for understanding climate change anxiety. *The role of parental identity in experiencing climate change anxiety and pro-environmental behaviors* explores climate anxiety and pro-environmental behaviors through the lens of parental identity (Pinho), illustrating how personally central roles may shape both emotional responses and engagement.

Role proximity is also explored in *Climate change anxiety in the scientific community: an exploratory study with Chilean climate change-related scholars* (Sapiains et al.), which examines climate change anxiety within a community that is professionally engaged with climate research, adding a perspective on how sustained informational exposure and responsibility may relate to distress and coping.

A qualitative perspective on coping and emotion negotiation is provided by *How Western Buddhist climate activists negotiate climate emotions* (Cairns and Pihkala), which analyzes how activists draw on religious and community practices to engage with a wide range of climate emotions (including anxiety, grief, anger, guilt, and compassion) while sustaining pro-environmental engagement.

### 2.4 From anxiety to action: behavioral pathways and collective engagement

The relationship between climate-related anxiety and pro-environmental behavior is neither conceptually simple nor empirically uniform. *Examining the relationship between ecological anxiety and pro-environmental behavior: personal and collective actions* contributes to this debate by differentiating personal and collective forms of action, thereby sharpening the behavioral phenomenology of ecological anxiety (Carasso Romano et al.). Together with the parental-identity perspective above, these findings encourage models that treat engagement as contingent on appraisals of efficacy and on realistic opportunities for meaningful individual and collective action.

### 2.5 Justice, structure, and the risk of depoliticizing distress

A recurring concern in climate anxiety scholarship is striking a balance between supporting individuals and recognizing that distress arises within structural conditions. *Climate anxiety as a call to global justice* explicitly frames climate anxiety within a justice-oriented perspective (Hanife et al.), cautioning against approaches that reduce the phenomenon to an individual deficit while downplaying inequality, responsibility, and political agency.

Similarly, the inclusion of an emissions-focused contribution (Liu et al.) serves as a reminder that the psychological consequences of climate change unfold alongside (and are shaped by) mitigation trajectories, land-use decisions, and broader socio-ecological systems.

## 3 Implications and future directions

Across the 17 contributions, several cross-cutting implications emerge. First, continued progress depends on specifying which facet is being measured (e.g., worry, anxiety symptoms, functional impairment, or broader emotion profiles) and ensuring that the instruments are psychometrically robust and culturally appropriate. Second, when climate change anxiety is linked to cognitive or functional outcomes, future research should prioritize designs that clarify temporal ordering and mechanisms (for example, the roles of rumination, attentional capture by threat cues, sleep disruption, and perceived controllability should be considered). Future research into the neurocognitive mechanisms that contribute to, and result from, climate anxiety may advance current neurocognitive models of climate anxiety and the role it plays in motivating climate action. Third, identity- and role-based perspectives suggest that climate-related distress may be intensified by personally central responsibilities (e.g., parenting) or by professional proximity (e.g., climate science), pointing to the value of supportive approaches that acknowledge moral emotions, meaning-making, and sustained engagement.

Finally, the justice-oriented work in this Research Topic underlines that supportive responses should not be confined to the individual level. Interventions and public communication that strengthen collective efficacy, foster realistic action pathways, and acknowledge unequal exposure and responsibility may be particularly important for reducing debilitating impairment while preserving the motivational potential of climate concern.

## 4 Conclusion

This Research Topic illustrates a maturing field: measurement is being refined across populations; construct boundaries are being debated explicitly; and the empirical lens is widening from affective distress to cognition, functioning, identity, coping, and justice. Taken together, this Research Topic supports an approach that validates climate-related distress as intelligible while remaining alert to functional costs and unequal burdens, and it charts clear priorities for rigorous, context-sensitive research and practice.

